# Vitamin D affects glucocorticoid action in target cells

**DOI:** 10.18632/oncotarget.13997

**Published:** 2016-12-16

**Authors:** Eva Kassi, Narjes Nasiri-Ansari, Athanasios G. Papavassiliou

**Affiliations:** Department of Biological Chemistry, Medical School, National and Kapodistrian University of Athens, Athens, Greece

**Keywords:** Vitamin D, glucocorticoids, glucocorticoid receptor, gene regulation, peripheral blood mononuclear cells, Neuroscience

It is known that administration of glucocorticoids is the first choice therapy in many autoimmune and inflammatory diseases. Glucocorticoid resistance is a major problem in the treatment of a variety of diseases since 30% of patients display it [1]. Observational studies have linked vitamin D deficiency with increased risk of various inflammatory and autoimmune maladies, while replenishing vitamin D appears to reduce the incidence of diseases and improve their course [2]. In this context glucocorticoids and vitamin D can be often co-administered in clinical practice albeit it remains unclear how administration of vitamin D could alter the response to glucocorticoids.

A wide range of experimental designs have been constructed aiming at studying the effects of vitamin D on immune responses; however, these experiments usually do not reflect the physiological conditions (i.e. concentrations that are not biologically attainable, time courses that do not correspond to physiological states).

We studied the effect of the long-term (11 days) repletion of vitamin D -the supplementation of which is often proposed for the prevention or even treatment of autoimmune / inflammatory diseases- on the sensitivity of peripheral blood mononuclear cells (PBMCs) to glucocorticoids [3].

We used the active metabolite of vitamin D, 1,25(OH)_2_D, at concentrations that are biologically achievable and co-incubated with dexamethasone for the last 48 hours at saturating dose regarding *glucocorticoid-induced leucine zipper* (*GILZ*) expression, a dexamethasone inducible gene that mediates mainly anti-inflammatory and immunosuppressive glucocorticoid actions in a variety of cell types. We found that long-term action of vitamin D downregulated *GILZ* expression thus inducing glucocorticoid resistance in PBMCs derived from healthy volunteers. Based on our results this effect can, at least in part, be attributed to: i) decrease of *glucocorticoid receptor* (*GR*) expression owing to a mechanism that engages histone deacetylase(s) (HDACs), and ii) inhibition of GR translocation to the nucleus via modulation of the phosphorylation state of GR; in fact, vitamin D decreased phosphorylation of GR at Ser211 whilst enhanced phosphorylation of GR at Ser203 [3].

The role of GR phosphorylations at Ser203 and Ser211 in the inhibitory effect of vitamin D remains to be definitively established, and the responsive mitogen-activated protein kinase (MAPK) signaling pathway (i.e. extracellular signal-regulated kinase (ERK), c-Jun N-terminal kinase (JNK), p38 cascades) should be identified. Furthermore, the class(es) and member(s) of HDAC implicated in the reduction of *GR* expression remain to be determined. Other mechanisms such as reduced glucocorticoid binding to GR due to nitrosylation of the receptor molecule, augmented P-glycoprotein activity, altered ligand availability due to altered corticosteroid-binding globulin (CBG) levels and/or decreased GR levels owing to microRNAs, should be investigated as candidates for the vitamin D-induced alterations in glucocorticoid responsiveness. Notably, according to unpublished data from our laboratory, incubation of PBMCs with vitamin D for 48 hours prior to dexamethasone treatment (6 hours) resulted in upregulation of *GILZ* expression, compared to dexamethasone alone, indicating that shorter incubation period with vitamin D elicited an opposite effect i.e. increased glucocorticoid sensitivity in healthy PBMCs.

These data are in concert with a previous report showing that short-term (21 hours) incubation of human PBMCs with vitamin D prior to dexamethasone treatment (3 hours) enhances dexamethasone induction of another GR responsive gene, *mitogen-activated protein kinase phosphatase-1* (*MKP-1*) [4]. Remarkably, the exposure duration appears to be an important determinant of the potency of vitamin D’s immunomodulatory effects [5]. It seems that there is a divergent interaction between vitamin D, glucocorticoids and their cognate receptors in relation to the duration of exposure to vitamin D, which remains inscrutable and of great clinical interest not only regarding autoimmune / inflammatory disorders but also other diseases treated with glucocorticoids, such as neoplasms. Glucocorticoids have been used in clinical oncology not only to alleviate side effects triggered by chemotherapy or radiotherapy but also to inhibit progression of different cancer types [6]. Moreover, recent studies associate vitamin D levels with the risk of various cancer types and with cancer disease prognosis [7, 8].

In view of the above, it becomes mandatory to explore and hopefully unveil the complexity in the interaction and cross-talk between the cell signaling pathways induced by these two steroid hormones since both can be co-administered in clinical practice. In addition, research efforts should aim to shed light on the mechanism via which vitamin D can modulate GR signaling in different cancer types, evaluating the expression of glucocorticoid-regulated genes that are implicated in tumor progression and/or inhibition. Until then, the various experimental conditions applied (pre- or co-incubation experiments, different cells, long- *vs* short-term incubations, different form/metabolite of vitamin D) should lead us to interpret with caution any relevant experimental data concerning the role of vitamin D in glucocorticoid sensitivity.
